# Imidazolium fumarate

**DOI:** 10.1107/S1600536809040793

**Published:** 2009-10-13

**Authors:** Rodolfo Moreno-Fuquen, Javier Ellena, Jahyr E. Theodoro

**Affiliations:** a Departamento de Química - Facultad de Ciencias, Universidad del Valle, Apartado 25360, Santiago de Cali, Colombia; b Instituto de Física de São Carlos, Universidade de São Paulo, USP, São Carlos, SP, Brazil

## Abstract

In the title compound, C_3_H_5_N_2_^+^·C_4_H_3_O_4_^−^, the dihedral angle between the imidazolium ring and the plane formed by the fumarate anion is 80.98 (6)°. In the crystal structure, inter­molecular O—H⋯O and N—H⋯O hydrogen bonds form extended chains along [100] and [01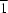], creating a two-dimensional network.

## Related literature

For background information on the anti-pyretic and anti-inflamatory biological activity of imidazole derivatives, see: Tudek *et al.* (1992 ▶); Puig-Parellada *et al.* (1973 ▶). For fumaric acid, see: Bednowitz & Post (1966 ▶) and for imidazole, see: McMullan *et al.* (1979 ▶). For hydrogen-bond motifs, see: Etter (1990 ▶). 
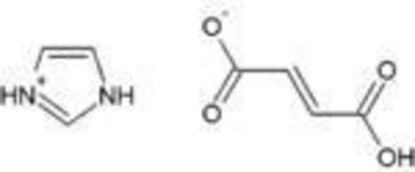


## Experimental

### 

#### Crystal data

C_3_H_5_N_2_^+^·C_4_H_3_O_4_^−^
*M* *_r_* = 184.15
Triclinic, 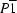

*a* = 7.4794 (4) Å
*b* = 7.7522 (3) Å
*c* = 8.4231 (4) Å
α = 69.695 (3)°
β = 81.415 (2)°
γ = 66.193 (2)°
*V* = 419.04 (3) Å^3^
*Z* = 2
Mo *K*α radiation
μ = 0.12 mm^−1^
*T* = 294 K
0.12 × 0.02 × 0.02 mm

#### Data collection

Nonius KappaCCD diffractometer
Absorption correction: none
7824 measured reflections
1922 independent reflections
1443 reflections with *I* > 2σ(*I*)
*R*_int_ = 0.087

#### Refinement

*R*[*F* ^2^ > 2σ(*F* ^2^)] = 0.051
*wR*(*F* ^2^) = 0.149
*S* = 1.07
1922 reflections
118 parameters
H-atom parameters constrained
Δρ_max_ = 0.31 e Å^−3^
Δρ_min_ = −0.29 e Å^−3^

### 

Data collection: *COLLECT* (Nonius, 2000 ▶); cell refinement: *SCALEPACK* (Otwinowski & Minor, 1997 ▶); data reduction: *DENZO* (Otwinowski & Minor, 1997 ▶) and *SCALEPACK*; program(s) used to solve structure: *SHELXS97* (Sheldrick, 2008 ▶); program(s) used to refine structure: *SHELXL97* (Sheldrick, 2008 ▶); molecular graphics: *ORTEP-3 for Windows* (Farrugia, 1997 ▶) and *Mercury* (Macrae *et al.*, 2006 ▶); software used to prepare material for publication: *PARST95* (Nardelli, 1995 ▶).

## Supplementary Material

Crystal structure: contains datablocks I, global. DOI: 10.1107/S1600536809040793/lh2920sup1.cif

Structure factors: contains datablocks I. DOI: 10.1107/S1600536809040793/lh2920Isup2.hkl

Additional supplementary materials:  crystallographic information; 3D view; checkCIF report

## Figures and Tables

**Table 1:** Hydrogen-bond geometry (Å, °)

*D*—H⋯*A*	*D*—H	H⋯*A*	*D*⋯*A*	*D*—H⋯*A*
O1—H1⋯O4^i^	0.82	1.75	2.5699 (16)	173
N2—H8⋯O3^ii^	0.86	1.82	2.6600 (18)	166
N1—H9⋯O4^iii^	0.86	1.94	2.7969 (16)	172

Symmetry codes: (i) 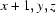; (ii) 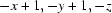; (iii) 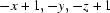.

**Fig. 1.**
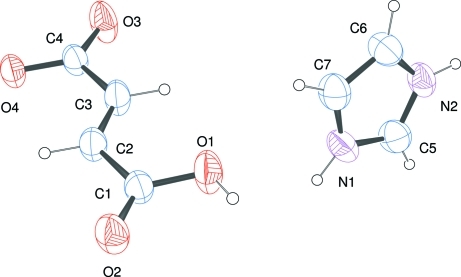
An ORTEP-3 (Farrugia, 1997) perspective drawing of (I), with the atomic labelling scheme for non-H atoms, which are represented by displacement ellipsoids drawn at the 50% probability level and, for the sake of clarity, H atoms are shown as spheres of arbitrary radius.

**Fig. 2.**
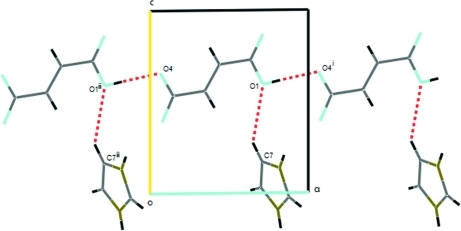
Part of the crystal structure of (I), showing the formation of C(7) chains running along [100] direction. Symmetry code: (i) x + 1, y, z; (ii) x - 1, y, z

**Fig. 3.**
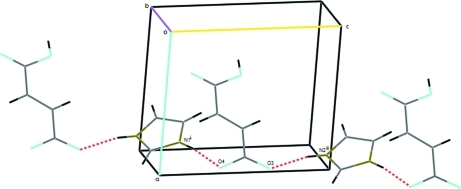
Part of the crystal structure of (I), showing the formation of C(8) chains running along [01–1]. Symmetry code: (i) -x + 1, -y, -z + 1; (ii) -x + 1, -y + 1, -z

## supplementary crystallographic information

### Comment

This work is part of a series of studies on the structural behavior of imidazole derivatives. Imidazole molecule is a heterocyclic compound which is present in important biological building blocks such as histidine, histamine, guanine (Tudek *et al.*, 1992). The analgesic, anti-inflammatory and anti pyretic properties of imidazole have been reported (Puig-Parellada *et al.*, 1973). In search of systems that form cyclic motifs linked by intermolecular hydrogen bonds, our research group focused its attention on the structural properties of molecular complex formed by fumaric acid (FUM) and imidazole (IM) moieties (I). The general crystallographic behavior of fumaric acid (Bednowitz & Post, 1966) and the imidazole free molecules (McMullan, *et al.*, 1979) may be used as reference systems in order to compare to the title imidazolium salt. A displacement ellipsoid plot of (I) with the atomic numbering scheme is shown in Figure 1. The free molecule of fumaric acid as well as other organic acids, are characterized by forming dimers in their structures. In the formation of the title adduct (I), dimers of fumaric acid decompose, to gain new and stronger hydrogen bonds and a more stable structure with imidazole moiety (see Table 1), (Nardelli, 1995). The C4–O4 bond length changes from 1.290 (5) in free fumaric acid molecule (Bednowitz & Post, 1966) to 1.2664 (17) Å in (I). The title structure shows a dihedral angle of 80.98 (6)° between the imidazole ring and the plane formed by FUM molecule. The other bond lengths and bond angles of (I) are in good agreement with the standard values and correspond to those observed in the free molecules. The transference of the proton from one of the carboxylic acid groups of FUM molecule to the basic N-atom of the IM molecule is carried out. This proton transfer allows the formation of new interactions in the FUM-IM adduct. The title adduct is characterized by the formation of hydrogen-bond interactions between O—H···O and N—H···O. One of the strongest hydrogen bond O—H···O interaction is responsible for crystal growth in [100] direction. Indeed, in a first substructure, atom O4 in the molecule at (*x*, *y*, *z*) acts as hydrogen bond donor to carboxyl O1^i^ atom in the molecule at (*x* + 1, *y*, *z*). The propagation of this interaction forms a C(7) (Etter, 1990) chain running along [100] direction (Fig. 2). In a second substructure, atom N2^ii^ in the molecule at (-*x* + 1, -*y* + 1, -*z*) acts as hydrogen bond donor to the atom O3 in the molecule at (*x*, *y*, *z*). While the atom N1^i^ in the molecule at (-*x* + 1, -*y*, -*z* + 1) acts as hydrogen bond donor to carboxy O4 atom in the molecule at (*x*, *y*, *z*). These interactions form C(8) chains which are running parallel to the [01–1] direction (Fig.3). All of these interactions in [100], and [01–1] directions define the overall two dimensional network.

### Experimental

The synthesis of the title adduct (I) was carried out by slow evaporation of equimolar quantities of fumaric acid (0.739 g, 637 mmol) and imidazole (0.433 g,) in 100 ml of aqueous solution of methyl alcohol at 2%. After 3 days, colourless prisms of poor quality, with a melting point greater than 424 K, were formed. Crystalline sample decomposes at temperatures greater than this value. The initial reagents were purchased from Aldrich Chemical Co., and were used as received.

### Refinement

All H-atoms were located from difference maps and were positioned geometrically and refined using a riding model with (C—H= 0.93, N—H= 0.86 and O—H= 0.82 A°) and *U*_iso_(H) (in the range 1.2–1.5 times *U*_eq_ of the parent atom).

### Crystal data

**Table:**

C_3_H_5_N_2_^+^·C_4_H_3_O_4_^−^	*Z* = 2
*M**_r_* = 184.15	*F*(000) = 192
Triclinic, *P*1	*D*_x_ = 1.460 Mg m^−^^3^
Hall symbol: -P 1	Mo *K*α radiation, λ = 0.71073 Å
*a* = 7.4794 (4) Å	Cell parameters from 4127 reflections
*b* = 7.7522 (3) Å	θ = 2.6–27.5°
*c* = 8.4231 (4) Å	µ = 0.12 mm^−^^1^
α = 69.695 (3)°	*T* = 294 K
β = 81.415 (2)°	Prism, colourless
γ = 66.193 (2)°	0.12 × 0.02 × 0.02 mm
*V* = 419.04 (3) Å^3^	

### Data collection

**Table:**

Nonius KappaCCD diffractometer	1443 reflections with *I* > 2σ(*I*)
Radiation source: fine-focus sealed tube	*R*_int_ = 0.087
graphite	θ_max_ = 27.5°, θ_min_ = 2.6°
CCD rotation images, thick slices scans	*h* = −9→8
7824 measured reflections	*k* = −10→10
1922 independent reflections	*l* = −10→10

### Refinement

**Table:**

Refinement on *F*^2^	Primary atom site location: structure-invariant direct methods
Least-squares matrix: full	Secondary atom site location: difference Fourier map
*R*[*F*^2^ > 2σ(*F*^2^)] = 0.051	Hydrogen site location: inferred from neighbouring sites
*wR*(*F*^2^) = 0.149	H-atom parameters constrained
*S* = 1.07	*w* = 1/[σ^2^(*F*_o_^2^) + (0.0832*P*)^2^ + 0.0432*P*] where *P* = (*F*_o_^2^ + 2*F*_c_^2^)/3
1922 reflections	(Δ/σ)_max_ < 0.001
118 parameters	Δρ_max_ = 0.31 e Å^−^^3^
0 restraints	Δρ_min_ = −0.29 e Å^−^^3^

### Special details

**Table:**

Geometry. All e.s.d.'s (except the e.s.d. in the dihedral angle between two l.s. planes) are estimated using the full covariance matrix. The cell e.s.d.'s are taken into account individually in the estimation of e.s.d.'s in distances, angles and torsion angles; correlations between e.s.d.'s in cell parameters are only used when they are defined by crystal symmetry. An approximate (isotropic) treatment of cell e.s.d.'s is used for estimating e.s.d.'s involving l.s. planes.
Refinement. Refinement of *F*^2^ against ALL reflections. The weighted *R*-factor *wR* and goodness of fit *S* are based on *F*^2^, conventional *R*-factors *R* are based on *F*, with *F* set to zero for negative *F*^2^. The threshold expression of *F*^2^ > σ(*F*^2^) is used only for calculating *R*-factors(gt) *etc*. and is not relevant to the choice of reflections for refinement. *R*-factors based on *F*^2^ are statistically about twice as large as those based on *F*, and *R*- factors based on ALL data will be even larger.

### Fractional atomic coordinates and isotropic or equivalent isotropic displacement parameters (Å^2^)

**Table:**

	*x*	*y*	*z*	*U*_iso_*/*U*_eq_	
O4	0.06367 (15)	0.14005 (15)	0.66024 (12)	0.0409 (3)	
O2	0.71272 (18)	0.03156 (19)	0.86710 (14)	0.0507 (4)	
O1	0.71448 (18)	0.2024 (2)	0.59326 (14)	0.0592 (4)	
H1	0.8224	0.1913	0.6157	0.089*	
O3	0.0768 (2)	0.3167 (2)	0.39170 (14)	0.0587 (4)	
N1	0.8197 (2)	0.25796 (19)	0.13620 (16)	0.0466 (4)	
H9	0.8473	0.1397	0.2063	0.056*	
N2	0.8081 (2)	0.4960 (2)	−0.09082 (16)	0.0443 (4)	
H8	0.8267	0.5605	−0.1937	0.053*	
C2	0.4385 (2)	0.1246 (2)	0.70462 (18)	0.0380 (4)	
H2	0.3770	0.0624	0.7974	0.046*	
C4	0.1475 (2)	0.2259 (2)	0.53479 (17)	0.0365 (4)	
C1	0.6358 (2)	0.1144 (2)	0.72978 (18)	0.0373 (4)	
C3	0.3458 (2)	0.2155 (2)	0.56046 (18)	0.0415 (4)	
H3	0.4073	0.2778	0.4678	0.050*	
C5	0.8781 (3)	0.3027 (2)	−0.0231 (2)	0.0475 (4)	
H5	0.9560	0.2119	−0.0781	0.057*	
C6	0.7006 (3)	0.5786 (2)	0.0293 (2)	0.0493 (4)	
H6	0.6342	0.7135	0.0147	0.059*	
C7	0.7089 (3)	0.4285 (3)	0.1717 (2)	0.0485 (4)	
H7	0.6500	0.4391	0.2751	0.058*	

### Atomic displacement parameters (Å^2^)

**Table:**

	*U*^11^	*U*^22^	*U*^33^	*U*^12^	*U*^13^	*U*^23^
O4	0.0376 (6)	0.0512 (6)	0.0305 (6)	−0.0243 (5)	−0.0008 (4)	0.0002 (5)
O2	0.0456 (7)	0.0681 (8)	0.0344 (6)	−0.0282 (6)	−0.0079 (5)	−0.0006 (5)
O1	0.0435 (7)	0.0886 (9)	0.0388 (7)	−0.0404 (7)	−0.0064 (5)	0.0083 (6)
O3	0.0548 (8)	0.0869 (9)	0.0302 (6)	−0.0419 (7)	−0.0100 (5)	0.0079 (6)
N1	0.0564 (9)	0.0408 (7)	0.0368 (7)	−0.0217 (7)	−0.0059 (6)	0.0006 (5)
N2	0.0499 (8)	0.0517 (8)	0.0288 (6)	−0.0259 (7)	−0.0019 (6)	−0.0012 (5)
C2	0.0358 (8)	0.0450 (8)	0.0334 (7)	−0.0207 (7)	0.0019 (6)	−0.0072 (6)
C4	0.0369 (8)	0.0428 (7)	0.0278 (7)	−0.0196 (6)	−0.0013 (6)	−0.0033 (6)
C1	0.0354 (8)	0.0427 (7)	0.0317 (7)	−0.0180 (6)	−0.0013 (6)	−0.0051 (6)
C3	0.0380 (8)	0.0550 (9)	0.0317 (7)	−0.0264 (7)	0.0010 (6)	−0.0042 (6)
C5	0.0506 (10)	0.0498 (9)	0.0401 (9)	−0.0181 (8)	−0.0011 (7)	−0.0127 (7)
C6	0.0584 (11)	0.0412 (8)	0.0431 (9)	−0.0182 (8)	−0.0011 (8)	−0.0079 (7)
C7	0.0554 (10)	0.0546 (9)	0.0341 (8)	−0.0233 (8)	0.0028 (7)	−0.0109 (7)

### Geometric parameters (Å, °)

**Table:**

O4—C4	1.2664 (17)	N2—H8	0.8600
O2—C1	1.2109 (18)	C2—C3	1.310 (2)
O1—C1	1.3084 (17)	C2—C1	1.489 (2)
O1—H1	0.8200	C2—H2	0.9300
O3—C4	1.2363 (17)	C4—C3	1.497 (2)
N1—C5	1.317 (2)	C3—H3	0.9300
N1—C7	1.356 (2)	C5—H5	0.9300
N1—H9	0.8600	C6—C7	1.339 (2)
N2—C5	1.307 (2)	C6—H6	0.9300
N2—C6	1.368 (2)	C7—H7	0.9300
			
C1—O1—H1	109.5	O2—C1—C2	121.36 (13)
C5—N1—C7	109.00 (14)	O1—C1—C2	114.43 (12)
C5—N1—H9	125.5	C2—C3—C4	124.42 (14)
C7—N1—H9	125.5	C2—C3—H3	117.8
C5—N2—C6	108.60 (13)	C4—C3—H3	117.8
C5—N2—H8	125.7	N2—C5—N1	108.58 (15)
C6—N2—H8	125.7	N2—C5—H5	125.7
C3—C2—C1	124.31 (14)	N1—C5—H5	125.7
C3—C2—H2	117.8	C7—C6—N2	107.01 (15)
C1—C2—H2	117.8	C7—C6—H6	126.5
O3—C4—O4	124.23 (14)	N2—C6—H6	126.5
O3—C4—C3	117.70 (13)	C6—C7—N1	106.81 (15)
O4—C4—C3	118.06 (12)	C6—C7—H7	126.6
O2—C1—O1	124.20 (13)	N1—C7—H7	126.6
			
C3—C2—C1—O2	178.47 (16)	C6—N2—C5—N1	−0.09 (19)
C3—C2—C1—O1	−1.1 (2)	C7—N1—C5—N2	−0.12 (19)
C1—C2—C3—C4	179.96 (13)	C5—N2—C6—C7	0.27 (19)
O3—C4—C3—C2	−179.96 (16)	N2—C6—C7—N1	−0.33 (19)
O4—C4—C3—C2	−0.5 (2)	C5—N1—C7—C6	0.3 (2)

### Hydrogen-bond geometry (Å, °)

**Table:**

*D*—H···*A*	*D*—H	H···*A*	*D*···*A*	*D*—H···*A*
O1—H1···O4^i^	0.82	1.75	2.5699 (16)	173
N2—H8···O3^ii^	0.86	1.82	2.6600 (18)	166
N1—H9···O4^iii^	0.86	1.94	2.7969 (16)	172

Symmetry codes: (i) *x*+1, *y*, *z*; (ii) −*x*+1, −*y*+1, −*z*; (iii) −*x*+1, −*y*, −*z*+1.
